# Why Medical Education Without Artificial Intelligence Still Matters: A Neuroscience-Informed Perspective

**DOI:** 10.2196/94594

**Published:** 2026-03-31

**Authors:** Charles Verdonk

**Affiliations:** 1 French Armed Forces Biomedical Research Institute, 1 rue du Général Valérie André, Brétigny-sur-Orge, 91220, France, +33 (0)1 78 65 12 70; 2 UMR VIFASOM, Université Paris Cité, Paris, France; 3 Laureate Institute for Brain Research, Tulsa, OK, United States

**Keywords:** artificial intelligence, medical education, cognitive off-loading, operational medicine, decision-making under stress

In their recent viewpoint, Izquierdo-Condoy et al [1] present a comprehensive analysis of the transformative potential, current applications, and future implications of artificial intelligence (AI) in medical education. They advocate for integrating AI literacy into medical curricula as health care systems become increasingly AI driven. I fully endorse this forward-looking perspective.

However, I would like to extend the discussion by highlighting a complementary yet underexplored issue: the implications of clinical practice in contexts where AI support is unavailable, unreliable, or deliberately restricted. While Izquierdo-Condoy et al [1] analyze the risks associated with integrating AI into medical curricula, less attention is given to the consequences of AI absence following training in AI-rich environments. The authors acknowledge that technological disparities may hinder equitable AI adoption [1], implying that AI-supported care cannot be universally guaranteed.

Emerging empirical evidence suggests that the removal of AI assistance after habitual use may adversely affect cognitive engagement and task performance. In an electroencephalography study, Kosmyna et al [2] reported that reliance on generative AI during essay writing was associated with reduced alpha- and beta-band functional connectivity—interpreted as diminished distributed network engagement—compared with unaided writing. Participants who initially relied on generative AI and were subsequently required to write without AI assistance exhibited persistently reduced connectivity and lower cognitive engagement, alongside poorer memory recall [2]. Behavioral performance decrements following AI withdrawal have also been documented in technical medical tasks, such as digestive endoscopy [3]. More broadly, the medical education literature cautions that when AI substitutes for clinical reasoning (cognitive off-loading) rather than augments it, risks to skill acquisition and retention may emerge [4]. Together, these findings suggest that sustained AI-mediated training may affect broader mechanisms of skill development, with vulnerabilities becoming evident in AI-absent contexts. Although preliminary, this evidence raises the possibility that prolonged AI reliance during training may reshape neurocognitive engagement in ways that have unintended consequences when independent performance is required.

These considerations are particularly salient in high-stakes, resource-constrained environments such as remote or isolated practices, military operations, disaster and humanitarian responses, and other extreme operational settings, where health care professionals’ cognitive performance is particularly strained by stress. In such contexts, the absence of AI support, combined with potential AI-mediated disruption of skill development during training, could compound vulnerability precisely when independent cognitive performance, such as clinical reasoning, is most critical.

This argument does not oppose AI integration. AI will likely enhance efficiency across many health care settings. However, ensuring safe and equitable care may require preserving AI-independent competence. Drawing inspiration from high-reliability industries such as aviation—where automation failure scenarios and minimum unaided practice are standard—medical education could incorporate structured AI-withdrawal exercises and defined thresholds of unaided proficiency [5]. In parallel, longitudinal research is needed to determine whether AI-mediated training produces durable changes in brain function and clinical performance.

As medical education accelerates toward AI integration, preparing physicians to practice both with and without AI support may represent a safeguard for patient safety and professional autonomy.

## Abbreviations

AI: artificial intelligence

## References

[1] Izquierdo-Condoy JS, Arias-Intriago M, Montero Corrales L, Ortiz-Prado E (2026). Artificial intelligence in medical education: transformative potential, current applications, and future implications. JMIR Med Educ.

[2] Kosmyna N, Hauptmann E, Yuan YT (2025). Your brain on ChatGPT: accumulation of cognitive debt when using an AI assistant for essay writing task. arXiv.

[3] Ho JCL, Qian Z, Lau LHS, Yip HC, Chiu PWY (2026). Artificial intelligence in digestive endoscopy training-the past, present, and future. Dig Endosc.

[4] Abdulnour REE, Gin B, Boscardin CK (2025). Educational strategies for clinical supervision of artificial intelligence use. N Engl J Med.

[5] Ong AY, Merle DA, Pollreisz A (2026). Flight rules for clinical AI: lessons from aviation for human-AI collaboration in medicine. NPJ Digit Med.

